# The Impact of Fullerenes as Doxorubicin Nano-Transporters on Metallothionein and Superoxide Dismutase Status in MCF-10A Cells

**DOI:** 10.3390/pharmaceutics14010102

**Published:** 2022-01-02

**Authors:** Natalia Zaręba, Klaudia Więcławik, Rene Kizek, Bozena Hosnedlova, Marta Kepinska

**Affiliations:** 1 Department of Pharmaceutical Biochemistry, Division of Biomedical and Environmental Analyses, Wroclaw Medical University, Borowska 211a St., 50-556 Wroclaw, Poland; natalia.zareba@umw.edu.pl; 2 Students Scientific Association, Division of Biomedical and Environmental Analyses, Faculty of Pharmacy, Wroclaw Medical University, Borowska 211a St., 50-556 Wroclaw, Poland; klaudia.wieclawik@student.umw.edu.pl; 3 CONEM Metallomics Nanomedicine Research Group (CMNRG), 116 36 Prague, Czech Republic; kizek@sci.muni.cz (R.K.); bozena.hosnedlova@post.cz (B.H.); 4 BIOCEV, First Faculty of Medicine, Charles University, Průmyslová 595, 25250 Vestec, Czech Republic

**Keywords:** fullerenes, drug delivery system, doxorubicin, epithelial cells, metallothionein, superoxide dismutase

## Abstract

This study aimed to synthesise C_60_–DOX complexes followed by the analysis of their effect on the concentration of metallothionein (MT) as a non-enzymatic antioxidant and on the concentration and activity of superoxide dismutase (SOD) as an antioxidant enzyme in healthy human mammary MCF-10A cells. Dynamic light scattering and electrophoretic light scattering were used to establish the size and zeta potential of the complexes. The MT and SOD concentrations were determined using the ELISA method; SOD activity was determined by tetrazolium salt reduction inhibition. Lower MT concentration following exposure of cells to both DOX and C_60_ fullerene compared to the control sample was found. However, the concentration of this protein increased as a consequence of the C_60_–DOX complexes action on MCF-10A cells compared to the control. C_60_ used alone did not affect the concentration and activity of SOD in MCF-10A cells. Application of free DOX did not activate cellular antioxidant defence in the form of an increase in SOD concentration or its activity. In contrast treatment of cells with the C_60_–DOX complex resulted in a decrease in SOD1 concentration and a significant increase in SOD activity compared to cells treated with free DOX, C_60_ and control. Thus, it was found that C_60_–DOX complexes showed potential for protective effects against DOX-induced toxicity to MCF-10A cells.

## 1. Introduction

The most widespread anthracycline is doxorubicin (DOX) [1,2]. DOX is administered in the treatment of organ cancers, especially breast cancer [3]. Treatment plans based on anthracycline antibiotics belong to the most effective therapies for breast cancer, which is the leading cause of death among women affected by cancer worldwide [4].

The DOX mechanism of action involves its binding to topoisomerase II and intercalating between base pairs of double DNA helix. This results in breaking the double-stranded DNA structure and inhibition of cellular growth [5,6]. DOX may also trigger an increased production of reactive oxygen species (ROS). ROS are produced intracellularly with the involvement of various mechanisms in such compartments as mitochondria, cytoplasm, endoplasmic reticulum, lysosomes, peroxisomes, and cell nuclei [7]. DOX oxidation via oxidoreductases to unstable semiquinone in the respiratory chain increases an already high level of ROS, resulting from the neoplastic process and the cells cross a threshold of apoptosis induction [8]. This mechanism (welcome in the context of cancer treatment) is also a mechanism of toxicity towards healthy cells of the body because of the lack of specificity. This drug toxicity towards tissues unaffected by the neoplasm is caused primarily by DOX-induced ROS production and long-term oxidative stress, leading to the cell structure destruction [8]. As a result, patients treated with cytostatics develop side effects such as cardiotoxicity, hepatotoxicity, gonadotoxicity and nephrotoxicity [5]. Under non-homeostatic conditions, such as a hypoxic and waste-laden tumour microenvironment, ROS damage may remove important oncogenic checkpoints through direct DNA damage. Because of their increased metabolic activity, cancerous cells show a constantly increased ROS level, activating signalling pathways and showing oncogenic activity, thus promoting cell proliferation, tumour progression and inactivating suppressor genes. Furthermore, ROS level increase in the cancerous cell is accompanied by an increase in the antioxidant ability to maintain homeostasis and avoid cell death [9,10,11].

**Figure 1:**
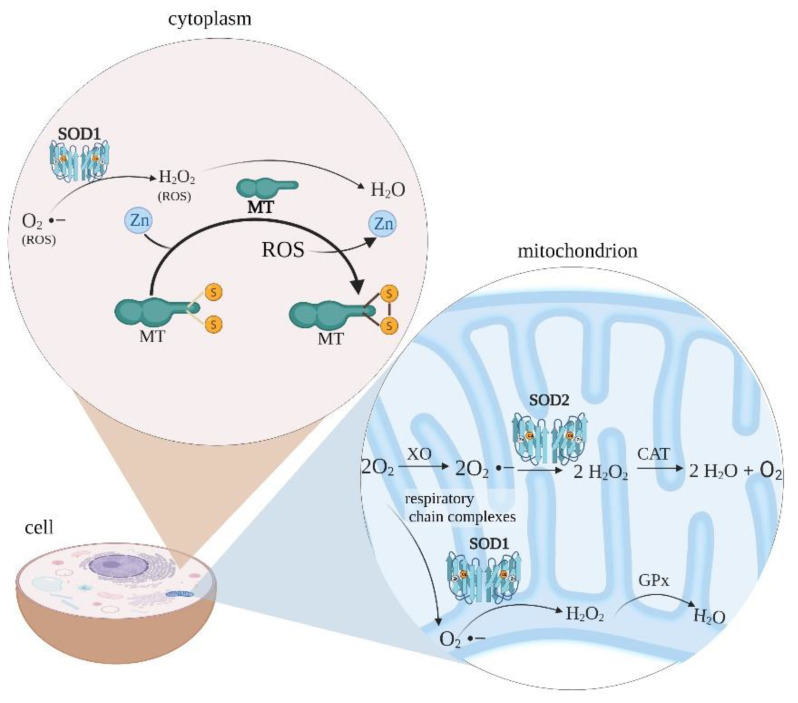
ROS management in the cell cytoplasm and mitochondrion. Metallothionein bound to zinc can scavenge free radicals because of its thiolate groups -SH. The Zn–MT complex provides a chemical basis on which the cysteine ligand exhibits antioxidant properties. In physiological terms, zinc bound to MT is released through oxidation of the thiolate group when the environment becomes oxidised, e.g., from the presence of free radicals. Creation of MT–disulfide follows zinc release. Mitochondrial-derived reactive oxygen species are formed by the conversion of oxygen to superoxide anion by xanthine oxidase or respiratory chain complexes in both matrix and intermembrane spaces. The superoxide anion is then converted to hydrogen peroxide by SOD. Hydrogen peroxide can be detoxified to water and oxygen with glutathione peroxidase or catalase. MT—metallothionein; SOD—superoxide dismutase; XO—xanthine oxidase; CAT—catalase; GPx—glutathion peroxidase; ROS—reactive oxygen species. The figure was created with BioRender.com.

The cell level of free radicals is related to the antioxidant system activity. Non-enzymatic molecules, such as metallothionein (MT), glutathione, and vitamins A, C, E, and enzymes, such as superoxide dismutase (SOD), catalase, and glutathione peroxidase, constitute a defence line against high intracellular ROS levels (Figure 1) [12].

Metallothionein is a metalloprotein representing non-enzymatic elements of the body’s antioxidant defence system [13]. An increased MT expression is thought to promote carcinogenesis and prevent tumour development in its initial stage. As an oncogenic protein, MT inhibits the activity of suppressor protein p53 and participates in angiogenesis of cancerous cells, affecting the expression of growth factors VEGF, FGF, NT, TGFα, and anti-apoptotic activity [14]. Furthermore, as a suppressor protein, MT detoxifies heavy metals (Hg, Cd), scavenges ROS, and protects DNA against potential damage [15]. On the other hand, an increased MT expression results in the accelerated tumour growth and a higher metastasis rate. The fact that MT overexpression results in higher resistance of cancerous cells to treatment (cytostatics, ionising radiation) is of the highest clinical significance [16]. Thus, the therapy may aim to reduce the MT level in cancerous cells without affecting its level in other tissues.

Superoxide dismutase has three isoforms in mammals. SOD1 is responsible for approximately 90% of SOD activity in a eukaryotic cell [17]. SODs are metalloenzymes that reduce oxygen radicals to the less toxic hydrogen peroxide, which is then broken down by appropriate catalases. It is known that an increased level and activity of antioxidant enzymes is a response to increased oxidative stress [18]. SOD1 shows overexpression in breast cancer [19]. In contrast to cancerous cells, the non-cancerous line shows a reduced level and activity of SOD1, indicating increased sensitivity of healthy cells to ROS compared to tumorigenic cells [20].

The issue of DOX toxicity towards normal cells may be solved by administering the drug together with compounds showing antioxidant properties, e.g., coenzyme Q10 [21]. Another way of avoiding DOX side effects is introducing DOX into transporters, e.g., nanoparticles, to deliver the drug directly to the specific target tissue and minimise normal cells damage [22].

Nanoparticles that combine these two properties are carbon-based fullerenes. The most common stable fullerene is C_60_ [23]. Thirty delocalised double bonds and 60 shared π electrons give the molecule aromatic properties and make it highly stable. The external nanoparticle surface enables various chemical reactions and the use of varied substituents. To improve solubility in water and biological systems, fullerene derivatives and polymers are created [24]. Numerous conjugated double bonds and an electrophilic character resulting from low unfilled molecular orbital make fullerenes antioxidative molecules [24]. Consequently, their application results in a low or zero toxicity towards tissues and cells. The low toxicity of these materials makes their use in humans and animals possible [25,26]. The use of fullerenes as drug nano-transporters was shown to have a positive influence on the therapy efficacy: it increases bioavailability, improves distribution processes, extends therapeutic index, and limits side effects, e.g., of melphalan [27], docetaxel [24,28], tamoxifen [29] and DOX [24,30]. Moreover, using DOX and the nano-transporter complex increases drug cytotoxicity towards the cancerous cell compared to healthy tissues [31].

The present study aimed to synthesise C_60_–DOX complexes and their characterisation by measuring the size and zeta potential, followed by the study of the effect of C_60_ used together with DOX on the MT-1/2 and concentration and activity of SOD in healthy human mammary MCF-10A cells.

## 2. Materials and Methods

### 2.1. Materials

An MCF-10A non-tumorigenic epithelial cell line (Cat. no. EP-CL-0525) was purchased from Elabscience. Fullerenes (Cat. no. 379646) and DOX (Cat. no. D1515) were obtained from Sigma-Aldrich (Darmstadt, Germany).

### 2.2. Methods

#### 2.2.1. Preparation of Fullerene–Doxorubicin Complexes

The preparation of complexes of fullerene with DOX was accomplished as previously published [31]. Briefly, a suspension of fullerenes in 4 mL of a HNO_3_ and H_2_SO_4_ mixture (1:3) was prepared. The suspension was heated in a water bath (Electrothermal, Birmingham, UK) at 70 °C for 7 h and then transferred to an ultrasonic bath for 30 min (Ultrasonic Cleaner T, SWR^®^, Castex, Bydgoszcz, Poland). For phase separation, the suspension was placed in a centrifuge for 30 min at 14,000× *g* (Centrifuge 351R, MPW, Warsaw, Poland), the supernatant was removed, and the pellet was washed with 500 µL H_2_O and centrifuged again for 30 min at 14,000× *g*. This step was repeated three times. The pellet obtained after the last centrifugation was suspended in 500 µL of a DOX solution, placed in an ultrasonic bath for 30 min and then centrifuged for 30 min at 14,000× *g*. The supernatant was removed and obtained complexes were washed twice with 500 µL H_2_O. C_60_–DOX and C_60_ alone were suspended in 1 mL of distilled water each and stored under refrigeration (4 °C).

#### 2.2.2. Size and Zeta Potential Measurements

C_60_ and C_60_–DOX size measurements were performed up to 1 week after the synthesis by dynamic light scattering in water at 25 °C using a Zetasizer analyser (Malvern Zetasizer Nano ZS, Malvern, UK). The analysis was made using the Malvern Instruments software. The medium and material refraction factors were assumed to be 1.33 and 1.96, respectively, viscosity 0.89 mPa*s, while the detection angle was 90°, and the wavelength was 633 nm. Dielectric constant was 3.61. The samples were diluted 1000-fold with distilled water to obtain a concentration that would allow reliable assessment. The samples were transferred into a cuvette (DTS0012, Malvern Panalytical, Malvern, UK) and placed in the analyser for measurement. For each sample, measures were taken three times. Each value was the average of all measurements.

The zeta potential was measured up to 1 week after the synthesis, in water at 25 °C, using a Zetasizer analyser (Malvern Zetasizer Nano ZS, Malvern, UK). The analysis was made using the Malvern Instruments software. The samples were prepared to correspond with the particle size measurement. Malvern cuvettes (DTS1070, Malvern Panalytical, Malvern, UK) were applied to take measures. Each measurement was performed three times. Each value was the average of all measurements.

#### 2.2.3. Fluorescence Measurements of DOX

To examine DOX concentrations in the prepared C_60_–DOX complexes, the samples were described by fluorescence measurements in a Wallac 1420 Victor 2 multilabel counter (PerkinElmer, Poway, CA, USA). The following concentrations of DOX standard solutions were prepared: 0.5; 2.5; 5; and 15 µM and placed on a Zell-Kontakt plate (Cat. no. 211601, Nörten-Hardenberg, Germany). Fluorescence was estimated at an excitation wave λ = 480 nm and an emission wave λ = 535 nm. The emission wave for DOX at which measurements should be performed is 590 nm, but because of the device’s capabilities the measure was performed at the emission wave λ = 535 nm and the study was based on these results.

#### 2.2.4. Culture and Preparation of MCF-10A Cells

A medium of the following composition was used to culture MCF-10A cells: DMEM/F12 medium (Dulbecco’s Modified Eagle’s Medium/Nutrient Mixture F-12 Ham, Cat. No. D8062, Sigma-Aldrich, Darmstadt, Germany), 5% Horse Serum (Cat. No. H1270, Sigma-Aldrich, Darmstadt, Germany), 20 µg/mL Recombinant Human EGF (Cat. No. PHG0311l, Gibco, ThermoFisher Scientific, Waltham, MA, USA), 0.5 µg/mL Hydrocortisone cortisol (Cat. No. H6909, Sigma-Aldrich, Darmstadt, Germany), 10 µg/mL Insulin solution human (Cat. No. I9278, Sigma-Aldrich, Darmstadt, Germany), 1% NEAA Mem Non-Essential Amino Acids (Cat. No. 11140-050, Gibco, Thermo Fisher Scientific, Waltham, MA, USA), 100 µg/mL cholera toxin from *Vibrio cholerae* (Cat. No. C8052, Sigma-Aldrich, Darmstadt, Germany), 1% gentamicin (Cat. No. 03-035-1C5, Biological Industries, Beit-Haemek, Israel). The presence of cells in the culture bottle was observed using a microscope (Opta-Tech MW50). After three cell passages, the medium was removed from the culture bottle and the cells were washed twice with phosphate-buffered saline (PBS, Cat. No. D8537, Sigma-Aldrich). A 0.25% trypsin solution (Cat. No. 15400054, ThermoFisher Scientific, Waltham, MA, USA) was added. The DMEM/F12 medium was then added, and thus, the trypsinisation process was stopped. The contents of the culture bottle were transferred to a Falcon tube and centrifuged at 20 °C for 10 min at 1500× *g* (Centrifuge MPW 351R). The supernatant was removed, the cell pellet was resuspended in cell culture medium. The cells were transferred into a Nest Biotechnology 96-well sterile plate, (Cat. No. 010921BL01) in such a way that 30,000 cells per well were present. The plates were incubated at 37 °C, 5% CO_2_ for 72 h. For treatment of cells with the respective agents, DOX, C_60_ and C_60_–DOX were diluted in culture medium. Control cells were not treated with additional agents and were cultured in pure culture medium.

#### 2.2.5. Cell Lysis

The culture medium was removed from the wells and the plate was washed with PBS. The Passive Lysis 5× Buffer (Promega E1941) was used to perform cell lysis. A total of 70 µL of 5× diluted buffer was added to each well. The lysis process was carried out for 20 min with continuous moderate agitation on a plate shaker (StatFax 2200 Avarness Technology Inc., Palm City, FL, USA) at room temperature until the cells detached from the plate surface. Lysates were pipetted off and centrifuged, while the obtained supernatant was placed in clean, labelled Eppendorf type tubes and stored in the freezer (−20 °C) for further analysis.

#### 2.2.6. Total Protein Concentration Measurement by the Bradford Method

Total protein concentration was determined by the Bradford method. Bradford reagent was prepared by dissolving 50 mg of Coomassie G—250 Brilliant Blue (Sigma Chem. Company, Darmstadt, Germany, Cat. No.: 18C-01320B-1131) in 1 mL of 96% ethanol (Stan Lab, Cat. No.: 603-002-00-5). After 24 h, the prepared mixture was combined with 24 mL of 96% ethanol and 50 mL of 85% orthophosphoric acid (Chempur, Piekary Śląskie, Poland, Cat. No.: 115691508), the whole mixture was made up to 100 mL with distilled water and mixed thoroughly. Then 20 µL of lysates was placed on a 96-well test plate (Beckman Coulter, Warsaw, Poland, Cat. No. 609844). Next, 200 µL of Bradford reagent was added to each well and the plate was incubated at room temperature. After 8 min, absorbance was measured at λ = 595 nm in a Multiskan GO spectrophotometer (ThermoFisher Scientific, Waltham, MA, USA).

#### 2.2.7. Metallothionein Concentration

The assay was performed according to the method described by Milnerowicz and Bizoń with modifications [32]. The direct quantitative enzyme-linked immunosorbent assay (ELISA) method was used to establish MT-1/2 concentration. The coated antigen was incubated with a primary antibody against MT: UC1MT (Cat. No.: MA1-25479, ThermoFisher Scientific, Waltham, MA, USA), diluted 1:2000. The next step was using a secondary antibody against the previously used primary antibody. Next, 100 μL of secondary goat anti-mouse polyclonal antibodies (Cat. No.: E0433, DakoCytomation, Glostrup, Denmark) was used, diluted 1:400. A diluted solution of HRP–avidin (horseradish peroxidase–avidin complex) (Cat. No.: P0347, DakoCytomation, Glostrup, Denmark) was added to the wells at a ratio of 1:800. The reaction was visualised by adding the ODP solution (Cat. No.: P-8287, Sigma-Aldrich, Darmstadt, Germany.) in 100 mL of 0.05 M phosphate citrate solution. Before use, 40 μL of 30% H_2_O_2_ (Cat. No.: 885195730, Avantor, Arnhem Netherlands) was added to the buffer. A 15-min incubation was performed. To stop the colour reaction, 50 μL of 3 M hydrochloric acid (Cat. No.: 805313164, Chempur, Piekary Śląskie, Poland) was added to all wells. Absorbance measurements were performed at 490 and 630 nm as a differential wavelength. MT concentrations were converted to the total protein concentration determined by the Bradford method.

#### 2.2.8. Concentration of SOD1 and Total SOD Activity

SOD1 concentration was determined using the commercial SOD1 Human ELISA Kit^®^ (Cat. No. BMS222, ThermoFisher Scientific, Waltham, MA, USA). In the assay, SOD1 in the sample and human SOD1 standards were bound by antibodies coated on the microwells. The introduction of a solution containing antibodies directed against antigens of the test protein, conjugated with horseradish peroxidase (HRP), produced a coloured product because of the addition of tetramethylbenzidine (TMB), a substrate for HRP. The reaction was stopped by the addition of phosphoric acid. Then the absorbance was measured at 450 nm. The concentration of SOD1 in the test samples was read and calculated from a standard curve. Next, the concentration of SOD 1 was converted to the total protein concentration in the samples.

SOD activity was established with a superoxide dismutase assay (Cat. No. 706002, Cayman, Ann Arbor, MI, USA) following manufacturer’s protocol. SOD activity is determined by the inhibition of the tetrazolium salt reduction reaction. One unit of SOD activity is defined as the amount of enzyme required to exhibit 50% dismutation of the superoxide radical. A standard curve was prepared using SOD standard dilutions. Briefly, SOD standards and samples were placed in the wells with a radical detector containing tetrazolium salt. The reaction was initiated by adding xanthine oxidase. After incubation absorbance was measured at 450 nm. SOD activity in the tested lysates was converted to the amount of total protein, called specific enzyme activity.

#### 2.2.9. Statistical Analysis

All the experiments were performed in triplicate. Statistical calculations were done using the Statistica 9.1 (StatSoft, Tulsa, OK, USA) software with the acquisition of mean values and standard deviation. The normality of the variables was analysed using the Shapiro–Wilk W test. Student’s *t*-test was used to evaluate the significance of the differences between groups. In all instances, *p* < 0.05 was considered statistically significant.

## 3. Results

### 3.1. Biophysical Characteristics of C_60_–DOX Complexes

**Figure 2:**
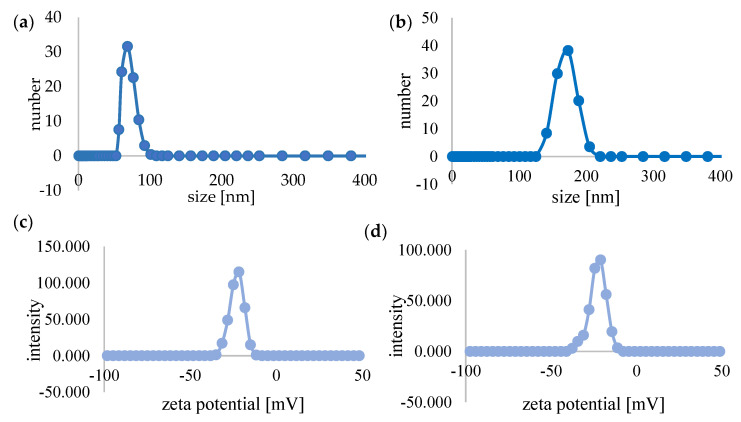
Biophysical characterisation of C_60_ and complexes of C_60_-DOX. Particle size distribution diagram of the C_60_ (**a**) and C_60_-DOX complexes (**b**). Zeta potential of C_60_ (**c**) and C_60_-DOX complexes (**d**).

The sizes of C_60_ and C_60_–DOX complexes were estimated by dynamic light scattering. Figure 2a,b shows the distribution of the number of light-scattering particles according to their hydrodynamic diameters in the studied system. The hydrodynamic diameter of C_60_ was 68.5 nm (Figure 2a), while the diameter of the complexes was larger and increased to 173 nm (Figure 2b).

Zeta potential of fullerenes and C_60_–DOX complexes was determined. The zeta potential of fullerenes was −21.6 mV, while the potential value of the complexes was −21 mV (Figure 2c,d).

### 3.2. The Effect of C_60_–DOX Complexes on Metallothionein Concentration in MCF-10A Cells

**Figure 3:**
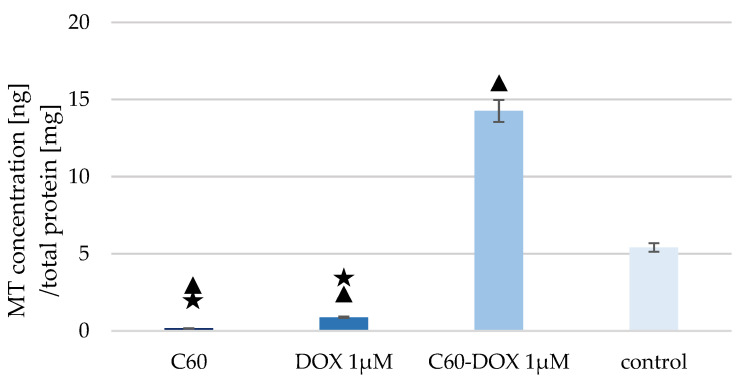
MT concentration in MCF-10A cells treated with C_60_, DOX and C_60_–DOX complex. The triangle marks a statistically significant difference compared with the control (*p* < 0.05). The asterisk marks statistically significant difference compared with the C_60_–DOX (*p* < 0.05).

The concentration of MT-1/2 in the lysate of cells treated with C_60_, DOX and complexes was measured (Figure 3).

A lower concentration of MT-1/2 in cells treated with both C_60_ and DOX was found compared to the control sample. However, a much higher concentration of MT-1/2 was recorded in the case of cells treated by C_60_–DOX complexes. MT concentration in cells treated with complexes increased almost 3-fold compared to the control sample. The concentration of MT was also higher in the sample with cells treated with C_60_–DOX complexes than in cells supplemented with C_60_ or free DOX.

### 3.3. The Effect of C_60_–DOX Complexes on SOD Concentration and Activity in MCF-10A Cells

**Figure 4:**
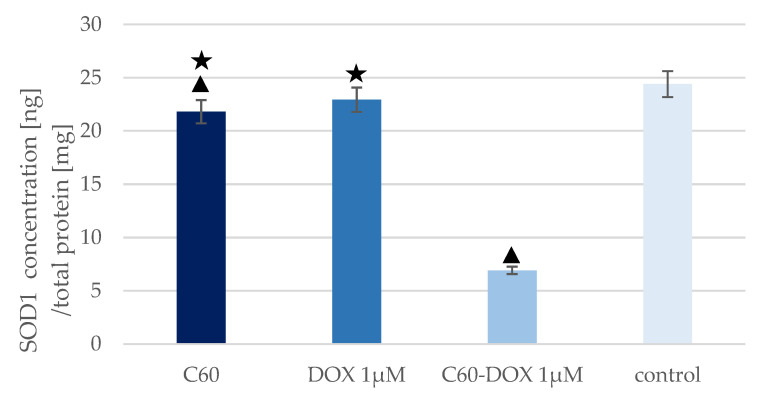
SOD1 levels in MCF-10A cells treated with C_60_, DOX and C_60_–DOX complexes. The triangle marks a statistically significant difference compared with the control (*p* < 0.05). The asterisk marks statistically significant differences compared with the C_60_–DOX (*p* < 0.05).

SOD1 levels in MCF-10A cells treated with C_60_, DOX, and C_60_–DOX were determined and converted to total protein concentration in cell lysates (Figure 4).

**Figure 5:**
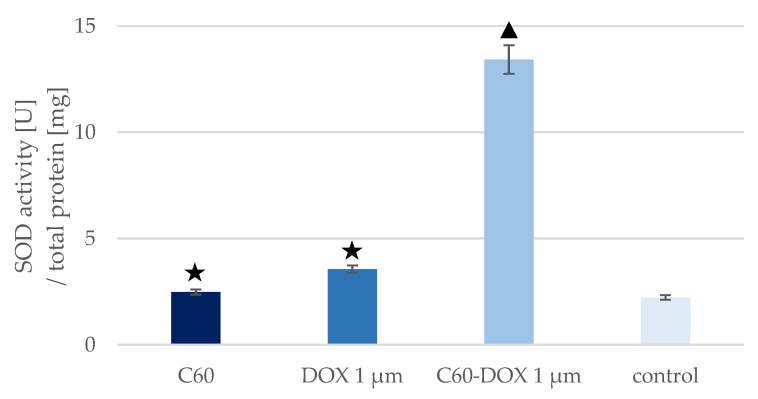
SOD activity in MCF-10A cells treated with C_60_, DOX and C_60_–DOX complexes. The triangle marks a statistically significant difference compared with the control (*p* < 0.05). The asterisk marks a statistically significant difference compared with the C_60_–DOX (*p* < 0.05).

The highest SOD1 concentration was observed in the control sample—MCF-10A cells without C_60_, drug or complexes. Cells treated with free DOX and cells treated with C_60_ showed a slight decrease in SOD1 concentration compared to the control sample, but only for C_60_ was the reduction statistically significant compared with the control. The greatest reduction in SOD1 concentration was noted in cells treated with C_60_–DOX complexes. The concentration of SOD1 in these cells was more than three times lower than in the control. In every analysed sample, the concentration of SOD1 was lower than in the control sample. The concentration of SOD1 was also lower in the sample with cells treated with C_60_–DOX complexes than in cells supplemented with C_60_ or free DOX. The highest SOD activity (Figure 5) was observed in cells treated with C_60_–DOX complexes. It was over six times higher than SOD activity in the control sample. SOD activity in cells treated with free DOX and cells treated with C_60_ was slightly higher than in the control but not statistically significant. The opposite was true for cells treated with C_60_–DOX complexes—SOD activity increased more than sixfold compared to the control samples. In cells treated with the nano-complex, SOD activity was also higher than when only the drug or C_60_ was added.

SOD activity per SOD1 concentration in MCF-10A cells treated with C_60_, free DOX and C_60_–DOX complexes is shown in Table 1.

Cells treated with C_60_–DOX complexes had the lowest SOD1 concentration and the highest SOD activity in relation to both total protein concentration and SOD1 concentration.

## 4. Discussion

DOX is a drug commonly appearing in primary recommendations and regimens for perioperative chemotherapy of breast cancer [33]. The potent side effects of DOX damage the non-cancerous cells. The toxicity of this drug on non-tumorigenic tissues is mainly due to the intracellular DOX-induced ROS production and the long-term oxidative stress induction, leading to the destruction of cell structures. On the other hand, in the case of cancer cells, increased DOX-induced ROS production is one of the mechanisms of drug cytotoxicity and is a desirable effect during chemotherapy. However, it should be noted that the success of therapy is determined by short-term elevated ROS, while chronically high ROS levels lead to chemoresistance and increased tumour proliferation [34]. In order to avoid DOX toxic side effects, the use of various types of drug carriers is increasingly being explored. C_60_ and its derivatives are biocompatible, show no toxic effects on normal tissues at low concentrations, and have potent antioxidant potential, thus they can be used efficiently as drug carriers [35]. Knowledge of the physicochemical properties and parameters of aggregates formed by C_60_ in aqueous media is essential to assess the bioactivity of the nanostructure and its potential for biomedical applications. The synthesised nano-complexes as well as free C_60_ were subjected to particle size and zeta potential measurements. The obtained zeta potential value agreed with literature data and indicated the existence of a negative charge on the surface of C_60_ particles in the aqueous solution [36]. The combination of C_60_ with DOX resulted in a slight shift of the potential peak towards zero (from −21.6 mV for C_60_ to −21 mV for the complex). In the literature data, information about larger values of the potential peak shift after the formation of complexes with DOX can be found. Prylutskyy et al. [36] obtained values of −28 mV for C_60_ and +45 mV for the C_60_–DOX complex, which were explained by the influence of positively charged DOX, but there are also publications reporting values close to those determined in the present work: zeta potential for C_60_ −30 mV, for the complex −24 mV [31], zeta potential for C_60_ −25.5 mV [37] and −32.5 mV for the complex [38]. By analysing the zeta potential values, the stability of the dispersion can be considered. A potential close to 0 indicates instability and the possibility of coagulation or flocculation, values between ±10 mV and ±30 mV are described as borderline stable, between ±30 and ±40 as moderately stable, and values between ±40 and ±60 indicate good stability of the dispersion, so the results obtained in this study can be described as a borderline stable system [39,40]. The measurement of C_60_ particle size by the DLS method showed that its size in an aqueous solution was about 70 nm. Literature data report that the size of C_60_ particles is usually in the range of 100 to 490 nm [31,38], but the size of aggregates can increase with time. For example, Brant noted an increase in particle diameter of about 20 nm after 2 months [37]. According to Mchedlov et al. [41], the dispersion of C_60_ in water is a hydrophobic colloid. The system is heterogeneous and polydisperse. The particle diameter can vary from a few nm to about 200 nm, confirming the validity of the results. The 173 nm size of the studied C_60_–DOX complexes was much larger than the C_60_ particle size, which indicated the successful combination of C_60_ and DOX and was consistent with the data presented in studies where the size of C_60_–DOX complexes was 132–278 nm [38,42].

The duality of using antioxidants as drug carriers or as part of anticancer therapy appears to be unclear and still needs to be studied. Current research says that, on one hand, angiogenesis (and thus cancer cell invasion) is inhibited by antioxidants but, on the other hand, intracellular antioxidant enzymes in cancer cells may increase cancer cell resistance to certain therapeutic regimens. Therefore, comparative studies of cell signalling in response to antioxidants for all cell types seems to be necessary to fully define the role of antioxidants in cancer therapy [43]. The antioxidant defence system against intracellular ROS consists of, among others, non-enzymatic molecules such as MT [42]. When human mammary gland MCF-10A cells are exposed to DOX, the MT concentration in the cells decreases by about 85% compared to the control sample. Gajewski et al. [44] studied the effect of exposure of non-neoplastic cells to DOX. The MCF-10A growth was inhibited and the apoptosis mechanisms in the cells were increased. Cells showed elevated ROS level and a time-dependent increase in oxidised DNA bases. A decrease in the expression of DNA repair enzymes was also observed in the same study. Of clinical relevance, the used concentration of DOX (0.1 µM) induced such high levels of oxidative stress in MCF-10A cells that cell proliferation was altered [44]. In a study by Aminipour et al. [45], the permeability of DOX was estimated in relation to the membrane potential present on the surface of human mammary gland MCF-10A and human breast cancer cells MCF-7, and it was shown that the cancerous cell line was more resistant to DOX exposure compared to the non-cancerous one. With the knowledge that the MCF-10A cell line is less resistant to DOX and more susceptible to membrane penetration compared to MCF-7 cells, it can be concluded that the significantly reduced MT level in response to drug exposures is related to the altered proliferation of MCF-10A cells and their overexposure to DOX-induced oxidative stress [45]. The lowest MT level in MCF-10A cells compared to the control sample were obtained following exposure to C_60_. The nano-transporter exhibits antioxidant properties and thus can act as a free radical scavenger, protecting cells from the adverse effects of oxidative stress. It probably decreases the concentration of MT, which is stimulated, among others, by increased ROS level. A similar relationship was observed in our previous study [42]. MCF-7 cancer cells were exposed to C_60_, and MT concentration remained at the same level or decreased (depending on C_60_ concentration) in relation to the control sample. C_60_ can reduce the side effects of anthracyclines while increasing their cytotoxicity [46]. In the present study, we investigated the impact of C_60_ complexing with DOX on MT level in MCF-10A cells. The result was an increase in the protein concentration in non-cancerous cells in response to exposure to the C_60_–DOX complex. Jing et al. conducted a study in which neonatal mouse cardiomyocytes were treated with different concentrations of ZnCl_2_: 100 and 50 µM. For both, there was an increase in MT expression. In the next stage of the study, 50 µM ZnCl_2_ was used to maintain the highest possible viability of the cardiomyocytes. The reason was the reduced survival of myocardial cells of MT +/+ mice when the dose was higher than 50 µM. The cells were then exposed to the drug for 24 h. DOX showed no adverse effects on MT +/+ cardiomyocytes. Elevated MT expression was shown to protect healthy cells from the adverse effects of DOX. The increased MT expression in relation to the MCF-10A cell line may indicate that the C_60_–DOX complex protects cells from the adverse effects of anthracycline by stimulating their antioxidant system [47].

MTs overexpression in cancer cells is mainly associated with poor prognosis, even though the protein plays an antioxidant role. By increasing angiogenesis, MTs affect tumour growth, cell proliferation and cell repair processes [48]. In our previous study, MT levels were measured after application of the C_60_–DOX complex at different concentrations: 25 mg/mL C_60_ + 1 µM DOX, 50 mg/mL C_60_ + 1 µM DOX, 50 mg/mL C_60_ + 2 µM DOX [42]. In the present study, MT expression increased approximately threefold compared to the control sample. Using the same concentration of anthracycline in studies on MCF-7 cells (1 µM) and 25 and 50 mg/mL C_60_, MT expression decreased with increasing fullerene concentration. The effects of fullerenes on healthy and cancer cells are different, which is desirable in therapy.

To protect themselves from the cytostatic drug and allow tumour growth, cancer cells show the high efficiency of antioxidant defence mechanisms, e.g., increased SOD expression. Using the the Western blot method, Papa et al. [19] showed that in cancerous breast cell lines (MCF-7, MDA-MB231, MDA-MB453, BT474 and BT20) SOD1 is overexpressed. Conversely, in the non-malignant breast epithelial cell line MCF-10A the baseline SOD1 level is lower. SOD activity determined in the non-neoplastic cell line was also found to be lower relative to the cancer cell line [19]. In the present study, we measured SOD activity in untreated MCF-10A cells and noted that the SOD activity level was comparable to the SOD activity in MCF-10A cells reported by Papa et al. (2.2 U/mg and approximately 3 U/mg, respectively). Our previous study showed that in DOX-treated MCF-7 breast cancer cells, SOD level and activity increased relative to the control sample [42]. The small changes in the concentration (1.5 ng/mg) and activity (1.3 U/mg) of this antioxidant enzyme in DOX-treated non-cancerous MCF-10A cells compared to the control sample in the present study may explain why non-cancerous cells are more exposed to ROS-induced toxic effects of the drug, i.e., oxidative stress, than cancer cells. In non-cancerous MCF-10A cells cultured in a medium supplemented with C_60_, no significant difference in SOD levels and activity was observed relative to the control sample, which was consistent with data presented by Prylutska et al. on the effect of C_60_ on SOD levels and activity in normal mouse heart and liver cells [35].

In this study, we investigated the effect of the application of C_60_–DOX complexes on SOD level and activity in MCF-10A cells. Application of the formed C_60_–DOX complexes to MCF-10A cells resulted in a significant increase in SOD activity. A more than 3-fold increase in the enzyme activity after application of the C_60_–DOX complex, compared to the application of free DOX, was reported. The values obtained in this work are the same as the results presented by Srdjenovic et al. [49], who studied the effectiveness of fullerenol (C_60_(OH)_24_) as an antioxidant agent against DOX-induced pulmotoxicity. In the above experiment, rats were treated with DOX and DOX together with fullerenol, then a lung tissue sample was collected in which enzyme activities, including SOD, were determined. In the DOX-treated sample, SOD activity decreased compared to the control, whereas when DOX was applied with fullerenol, no decrease in SOD activity was observed [49]. Yeh et al. also considered the possibility of a protective effect of the antioxidant compound doxycycline on DOX-induced oxidative and apoptotic effects in mouse testes [50]. The results obtained in the present study appeared to be consistent with those reported by Yeh et al., where the testes of DOX-treated mice showed impaired spermatogenesis and the cells possessed reduced antioxidant activity expressed by reduced SOD activity. Similar to the treatment of C_60_–DOX on MCF-10A cells, higher SOD activity was observed in the testes of mice after treatment with DOX in combination with the antioxidant doxycycline, compared to treatment with DOX alone. This indicated that the use of additional antioxidants, e.g., C_60_, may effectively prevent the DOX-induced decrease in SOD activity. This beneficial phenomenon is probably related to the ability of C_60_ to act as an ROS scavenger or SOD mimetic and reduce ROS production to optimal level for activation of antioxidant enzymes. High SOD activity has been shown to increase cell survival in the presence of DOX through its role as a free radical scavenger [35].

In addition to the enzymatic activity, SOD concentration in the tested material was also determined. In lysate of MCF-10A cells treated with C_60_–DOX complex, high SOD activity was accompanied by low enzyme concentration compared to DOX-treated cells and control sample. The results of the measurements differed from those reported by Yeh et al., which indicated an increased expression of SOD1 compared to the free DOX treatment and the control sample [50]. In both studies, the concentration was determined by Cu/Zn-specific SOD by ELISA. Yeh et al. [50] indicated that Cu/Zn SOD (SOD1) is the predominant SOD isoform in testicular tissue, whereas for the breast gland cells tested, the ratio of the different SOD isoforms is not clearly defined in the literature. The obtained results of low SOD1 expression in the sample with simultaneous high SOD activity may be because the applied ELISA test detects the SOD1 isoform quantitatively, while the measurement of SOD activity includes both the cytoplasmic (SOD1) and mitochondrial (SOD2) fractions. Moreover, the discrepancies between the concentration and activity of SOD in MCF-10A cells treated with the C_60_–DOX complex may indicate that C_60_ improves the availability of enzyme active sites and thus acts as an inducer of activity. The antioxidant properties of C_60_ and its derivatives, proven in cell lines and animal models, result from the ability of these structures to accept up to six electrons. The mechanism of antioxidant activity was analysed using the example of a C_60_ derivative with three molecules of tris-malonic acid. The reaction between the C_60_ derivative and the superoxide occurred via catalytic superoxide dismutation, as indicated by oxygen regeneration, superoxide production and the lack of modification in the C_60_ derivative structure, among others. In a study led by Ali et al., it was demonstrated that treatment of mice lacking SOD2 expression with a C_60_ derivative prolonged their life significantly by acting as a biologically effective SOD mimetic [51]. SOD demonstrates increased synthesis during oxidative stress—superoxide radical is one of the factors indirectly inducing SOD gene expression. Therefore, it can be speculated that the reduced SOD concentration in cells treated with the complexes could be a response to the SOD-mimetic, antioxidant, free radical-lowering effect of C_60_ under DOX-induced oxidative stress. On the other hand, the recorded high SOD activity in MCF-10A cells with the addition of the C_60_–DOX complex could have been influenced by the cumulative effect of the antioxidant action of C_60_ and the more easily accessible active sites of the enzyme. In view of the results not being unequivocally confirmed in the available literature, a broader study should be carried out on this aspect. Nevertheless, the lower concentration of the enzyme accompanied by higher activity indicated that the combination of the nanomaterial with the drug shows high efficacy in the context of a potentially protective effect on healthy cells during DOX treatment, which, in combination with the previous results published by our group, suggests the high therapeutic potential of C_60_–DOX complexes in cancer therapy, including breast cancer.

## 5. Conclusions

The use of C_60_ in DOX therapy affects the efficiency of the antioxidant system in non-cancerous cells. Exposure of MCF-10A cells to C_60_ results in an intracellular decrease in MT concentration above 30-fold (5.41 and 0.17 ng/mg, for control and C_60_ sample, respectively). Application of C_60_–DOX complexes in the therapy alters the expression of MT by increasing its concentration in MCF-10A cells over 2.5 times (5.41 and 14.26 ng/mg, for control and C_60_-DOX sample, respectively) and counteracting the adverse effects of DOX on healthy cells. The C_60_–DOX complexes cause an approximately sixfold increase in SOD activity in MCF-10A cells (2.23 and 13.42 U/mg, for control and C_60_–DOX samples, respectively), indicating the potential of the complexes to have a protective effect on healthy cells against DOX-induced toxicity.

It can be concluded that the nanomaterial–drug combination shows high efficacy in the context of a potentially protective antioxidant effect on healthy cells during DOX treatment. According to the obtained results and previous studies performed on cancerous MCF-7 cells, it suggests a high therapeutic potential of C_60_–DOX complexes in cancer therapy, including breast cancer.

## Figures and Tables

**Table 1:** SOD concentration and activity per total protein concentration and SOD activity per SOD1 concentration in MCF-10A cells treated with C_60_, DOX and C_60_–DOX complexes.

Sample	SOD1 Concentration [ng/mg Total Protein]	SOD Activity [U/mg Total Protein]	SOD Activity/SOD1 Concentration [U/ng]
Control	24.402	2.226	0.091
C_60_	21.807	2.483	0.114
DOX	22.931	3.557	0.155
C_60_–DOX	6.913	13.424	1.942

## Data Availability

Not applicable.
